# Barley stripe mosaic virus-mediated somatic and heritable gene editing in barley (*Hordeum vulgare L*.)

**DOI:** 10.3389/fpls.2023.1201446

**Published:** 2023-06-19

**Authors:** Suriya Tamilselvan-Nattar-Amutha, Stefan Hiekel, Franziska Hartmann, Jana Lorenz, Riddhi Vijay Dabhi, Steven Dreissig, Goetz Hensel, Jochen Kumlehn, Stefan Heckmann

**Affiliations:** Leibniz Institute of Plant Genetics and Crop Plant Research (IPK) OT Gatersleben, Seeland, Germany

**Keywords:** CRISPR/Cas9, BSMV, VIGE, albostrians, barley, genome editing

## Abstract

Genome editing strategies in barley (*Hordeum vulgare L.*) typically rely on *Agrobacterium*-mediated genetic transformation for the delivery of required genetic reagents involving tissue culture techniques. These approaches are genotype-dependent, time-consuming, and labor-intensive, which hampers rapid genome editing in barley. More recently, plant RNA viruses have been engineered to transiently express short guide RNAs facilitating CRISPR/Cas9-based targeted genome editing in plants that constitutively express *Cas9*. Here, we explored virus-induced genome editing (VIGE) based on barley stripe mosaic virus (BSMV) in *Cas9*-transgenic barley. Somatic and heritable editing in the *ALBOSTRIANS* gene (*CMF7*) resulting in albino/variegated chloroplast-defective barley mutants is shown. In addition, somatic editing in meiosis-related candidate genes in barley encoding ASY1 (an axis-localized HORMA domain protein), MUS81 (a DNA structure-selective endonuclease), and ZYP1 (a transverse filament protein of the synaptonemal complex) was achieved. Hence, the presented VIGE approach using BSMV enables rapid somatic and also heritable targeted gene editing in barley.

## 1 Introduction

RNA-guided clustered regularly interspaced short palindromic repeats (CRISPR)-associated (Cas) endonucleases emerged as a versatile tool for targeted genetic engineering in plants (Bao et al., 2019; Chen et al., 2019; Koeppel et al., 2019; Hinge et al., 2021; Hisano et al., 2021). In barley, the transfer of genetic reagents required to elicit Cas9-mediated targeted genetic engineering relies on stable genetic transformation (Lawrenson et al., 2015; Gerasimova et al., 2020). However, only a limited number of genotypes is amenable to efficient genetic transformation (Hensel et al., 2008; Yeo et al., 2014; Hoffie et al., 2021) and the isolation of stable genetic transformants is time- and labor-consuming. Hence, the rapid application of targeted genetic engineering in barley is hampered.

More recently, virus-induced genome editing (VIGE) emerged as a targeted genome editing tool for plants (Cody and Scholthof, 2019; Oh et al., 2021; Varanda et al., 2021; Gentzel et al., 2022; Uranga and Daròs, 2022). Plant viruses are engineered to deliver either guide RNAs into plants stably expressing endonucleases or even the complete genome engineering components (Cody and Scholthof, 2019; Metje-Sprink et al., 2019; Tsanova et al., 2021; Varanda et al., 2021; Gentzel et al., 2022). VIGE has been applied in dicot plants including *Nicotiana benthamiana, Arabidopsis thaliana*, and *Glycine max* using plant RNA viruses such as Tobacco rattle virus (TRV) (Ellison et al., 2020; Nagalakshmi et al., 2022), Pea early browning virus (PEBV) (Ali et al., 2018), Beet necrotic yellow vein virus (BNYVV) (Jiang et al., 2019), Potato virus X (PVX) (Ariga et al., 2020), Barley yellow striate mosaic virus (BYSMV) (Gao et al., 2019) or Sonchus yellow net rhabdovirus (SYNV) (Ali et al., 2015; Zaidi and Mansoor, 2017; Cody and Scholthof, 2019; Ellison et al., 2020; Ma et al., 2020; Nagalakshmi et al., 2022). In monocots, Foxtail mosaic virus in maize (FoMV) (Mei et al., 2019) or BSMV in wheat, cotton, and maize (Hu et al., 2019; Li et al., 2021; Chen et al., 2022a; Chen et al., 2022b; Wang et al., 2022) were employed for VIGE.

BSMV is a positive-sense RNA hordeivirus with a tripartite genome consisting of RNA α, β, and γ (Petty et al., 1990; Jackson et al., 2009). BSMV was harnessed to deliver sgRNAs into plants that ectopically express *Cas9*, such as monocots (wheat and maize) and the dicot *N. benthamiana*, for eliciting Cas9-mediated targeted genome editing in somatic tissues (Hu et al., 2019; Li et al., 2021; Chen et al., 2022a; Wang et al., 2022). In wheat, different frequencies of BSMV-mediated heritable gene editing were observed, ranging from 0.8 to 3.0% (Chen et al., 2022a; Chen et al., 2022b), 12.9 to 100% (Li et al., 2021), and 0 to 19% (Wang et al., 2022), depending on the genotype, type of sgRNA (with/without mobile RNA elements), and the target site (Notaguchi et al., 2014; Zhang et al., 2016; Ellison et al., 2020; Nagalakshmi et al., 2022).

BSMV also infects several other agronomically important cereal crops such as oats (*Avena sativa*) (Pacak et al., 2010), culinary ginger (*Zingiber officinale*) (Renner et al., 2009), rye (*Secale cereale*) (Groszyk et al., 2017), millet (*Setaria italica*), and barley (Jackson et al., 2009). In barley, BSMV infects several genotypes/cultivars (Edwards and Steffenson, 1996; Holzberg et al., 2002; Bruun-Rasmussen et al., 2007), has been harnessed for virus-induced gene silencing (VIGS) (Holzberg et al., 2002; Yuan et al., 2011; Lee et al., 2012; Dommes et al., 2019), enters the germline (Carroll, 1972; Carroll and Mayhew, 1976; Brlansky et al., 1986), and can be transmitted *via* grains (Carroll, 1972). Hence, these features offer an opportunity for efficient heritable editing in barley based on BSMV-mediated VIGE (BSMVIGE).

We explored BSMV as a VIGE tool in barley cv. Golden Promise plants expressing *Cas9* by targeting, as proof of principle, the *ALBOSTRIANS* gene, *CMF7*, involved in chloroplast development (Li et al., 2019). Somatic *cmf7* mutations, induced in virus-infected plants, can be transmitted to subsequent generations resulting in albino or variegated plants defective for *CMF7*. In addition, somatic mutations were induced in three meiosis-related genes in barley: *ASY1*, encoding for a meiotic chromosome axis-localized HORMA domain protein (Armstrong et al., 2002; Steckenborn et al., 2023), *MUS81*, encoding for a DNA structure-selective endonuclease involved in the formation of meiotic class II crossover (CO) (Hartung et al., 2006; Berchowitz et al., 2007; Higgins et al., 2008) as well as *ZYP1*, encoding for a transverse filament protein of the synaptonemal complex (SC) (Barakate et al., 2014; Steckenborn et al., 2023). Hence, BSMVIGE enables rapid somatic and heritable targeted gene editing in barley.

## 2 Materials and methods

### 2.1 Plant material and growing conditions

*N. benthamiana* plants were grown in a greenhouse at 22°C, relative humidity of 65%, and a photoperiod of 16/8h light/dark at 80-100 µmol m^-2^ s^-1^. Within a containment facility of biosafety level S2, after BSMV infection, plants were incubated at constant 24.5°C, relative humidity of 65%, and a photoperiod of 16/8h light/dark at 80-100 µmol m^-2^ s^-1^ in a growth cabinet (Polyklima). Barley plants expressing *Cas9* of *Streptococcus pyogenes* were grown in a greenhouse at constant 19°C, 65% relative humidity, and a photoperiod of 16/8h light/dark at 160-250 µmol m^-2^ s^-1^. Five days before and after the BSMV infection, plants were incubated in a controlled growth chamber (at constant 24.5°C, 65% relative humidity, and a photoperiod of 16/8h light/dark at 160-250 µmol m^-2^ s^-1^) within a containment facility of biosafety level S2. Offspring from virus-infected plants were grown under the same controlled conditions except at constant 21°C. Barley plants were supplemented with 0.2% Wuxal fertilizer (MANNA GmbH, Germany) once per week.

### 2.2 Construction of transformation vectors

An expression unit consisting of the maize *Ubi1* promoter and 5’-UTR including its intron 1, *Streptomyces pyogenes Cas9* with maize codon usage preceded by a 4x FLAG tag and bordered by two copies of the Simian virus 40 nuclear localization signal (NLS) was assembled in front of the *nopalin synthase* polyadenylation signal of *Agrobacterium tumefaciens* in plasmid pUbi-ABM (DNA Cloning Service, Germany). This unit was then transferred as a compatible SfiI fragment to the generic binary vector p7intUbi (DNA Cloning Service, Germany) to generate pSH151.

### 2.3 Generation of transgenic plants ubiquitously expressing *Cas9*

Transgenic barley plants were generated as described (Hensel et al., 2009; Marthe et al., 2015). Briefly, dissected immature embryos were inoculated and co-cultivated with the *A. tumefaciens* strain AGL1 (Lazo et al., 1991) carrying binary vector pSH151. After callus induction and plant regeneration under selective conditions using timentin to remove residual agrobacteria and bialaphos for transgenicity, plantlets with developed roots were transferred to the soil substrate. Transgenicity of regenerants (presence of *Cas9*) and *Cas9* expression were confirmed (for primer sequences see **Supplementary Table 1**). From two selected T_0_ plants based on *Cas9* expression and being phenotypically indistinguishable from the WT, homozygous progeny were produced by microspore-derived plant regeneration (Kapusi et al., 2013; Lippmann et al., 2015). From the doubled haploids obtained, progeny of line BG710-DH62 referred to as *ZmUBI::Cas9* plants (positive for *Cas9* expression) were used for BSMV infections.

### 2.4 Design of SpCas9-compatible guide RNAs

According to criteria defined by (Schindele et al., 2020), using CRISPOR (Haeussler et al., 2016) and RNAfold (Gruber et al., 2008), single guide (sg)RNAs with high target specificity, low off-target scores, and appropriate secondary structure were selected: CTCCTGGATTCAGGATCCAT(GGG), GCAGACGTTGCGGTAGGCGT(TGG), and TTCTAGATCAGACTTCACCG(AGG) complementary to a target region within exon 4 of *HvASY1* (HORVU.MOREX.r3.5HG0494140), exon 1 of *HvMUS81* (HORVU.MOREX.r3.3HG0257160) and exon 2 of *HvZYP1* (HORVU.MOREX.r3.2HG0172550), respectively. As controls, sgRNAs CTCAAGGCGTGGTATGACAG(AGG) and GGCGAGGGCGATGCCACCTA(CGG) specific to *HvCMF7* (HORVU.MOREX.r3.7HG0728080) (Li et al., 2019) and *AvGFP* (Wang et al., 2017) were used, respectively.

### 2.5 Generation of the generic guide RNA expression vector BSMV-γ-sg and its specification for Cas9 target motifs

**Figure 1:**
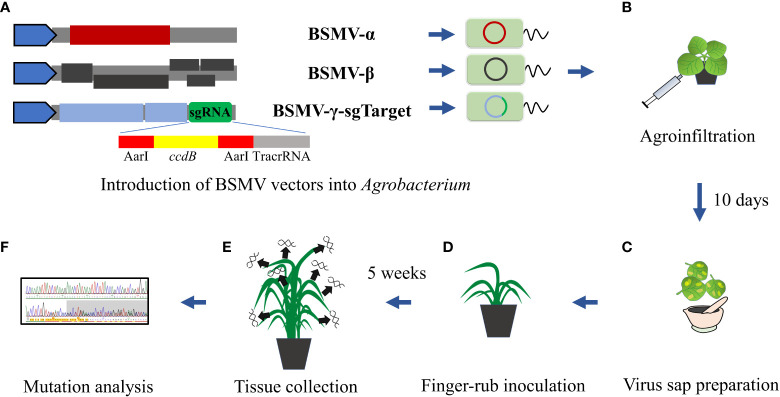
Schematic BSMVIGE workflow in barley. **(A)** Introduction of BSMV-α, BSMV-β, and BSMV-γ-sg vectors into *Agrobacterium*; **(B)** agroinfiltration using resuspended *Agrobacterium* mixtures harboring BSMVIGE vectors into *N. benthamiana* leaves; **(C)** virus leaf sap preparation 10 dpi; **(D)** finger-rub inoculation of virus sap into 3-4 week old *ZmUBI::Cas9* plants; **(E)** tissue collection from multiple somatic structures 5 wpi; **(F)** mutation analysis of Sanger reads using ICE synthego tool (Conant et al., 2022).

To generate BSMV-γ-sg enabling the expression of sgRNAs downstream of the γb ORF in RNAγ, a 359 bp sequence flanking the ligation-independent cloning (LIC) site in the pCa-LICγb plasmid (Yuan et al., 2011) was replaced with a 1065 bp custom synthesized sequence *via* HpaI/BamHI. This sequence consists of the BSMV-γb cDNA sequence, the *ccdB* gene flanked on both sites by AarI for insertion of target sequences, and the sgRNA scaffold sequence compatible with SpCas9 (**Figure 1A**, **Supplementary Table 2**). Annealed complementary sgRNA oligonucleotides were cloned into BSMV-γ-sg *via* Golden Gate cloning (Engler et al., 2008) using AarI and confirmed by Sanger-Sequencing employing BSMV-γ-sg insert-specific primers (**Supplementary Table 1**).

### 2.6 Plant infection

BSMV-α, BSMV-β, and BSMV-γ-sg vectors (Yuan et al., 2011) were introduced individually into *A. tumefaciens* strain AGL1 (**Figure 1A**). An equal mixture (optical density at 600 nm of 1.2 of each culture) of AGL1 carrying the three BSMV components was infiltrated into leaves of 21 days old *N. benthamiana* plants to generate a high titer of functional BSMV (**Figure 1B**). Virus-symptom spotted leaves were harvested after 10-12 days post-infection (dpi) and ground in 10 mM phosphate buffer (pH 7) containing 0.5% of each celite 545 (Roth) and silicon carbide (400 mesh particle size, Sigma-Aldrich) (**Figure 1C**) on ice. The virus sap was used to inoculate 3 to 4 weeks-old barley plants by finger-rub inoculation of fully-emerged second and third leaves (**Figure 1D**).

### 2.7 Confirmation of BSMV infection

OneTaq one-step RT-PCR kit (NEB) and target-specific primers (**Supplementary Table 1**) were employed to amplify a region spanning the sgRNA in BSMV-γ-sg using 10 ng of extracted total RNA from leaf tissue.

### 2.8 Detection of targeted genome editing

Target regions were amplified using target-specific primers (**Supplementary Table 1**) and purified amplicons were Sanger-sequenced (Eurofins Genomics) (**Figure 1E**). Mutation efficiency (InDel percentage at target sites) and mutation frequency (frequency of plants showing mutation efficiency of at least 5% for each target) were estimated from Sanger reads using the ICE Synthego web tool (Conant et al., 2022) (**Figure 1F**). Sanger chromatograms of selected M1 plants (examples for high, medium or no editing at corresponding target site) are shown in **Supplementary Figure 1**.

## 3 Results

### 3.1 BSMVIGE mediates somatic editing at the *ALBOSTRIANS* locus in barley

**Figure 2:**
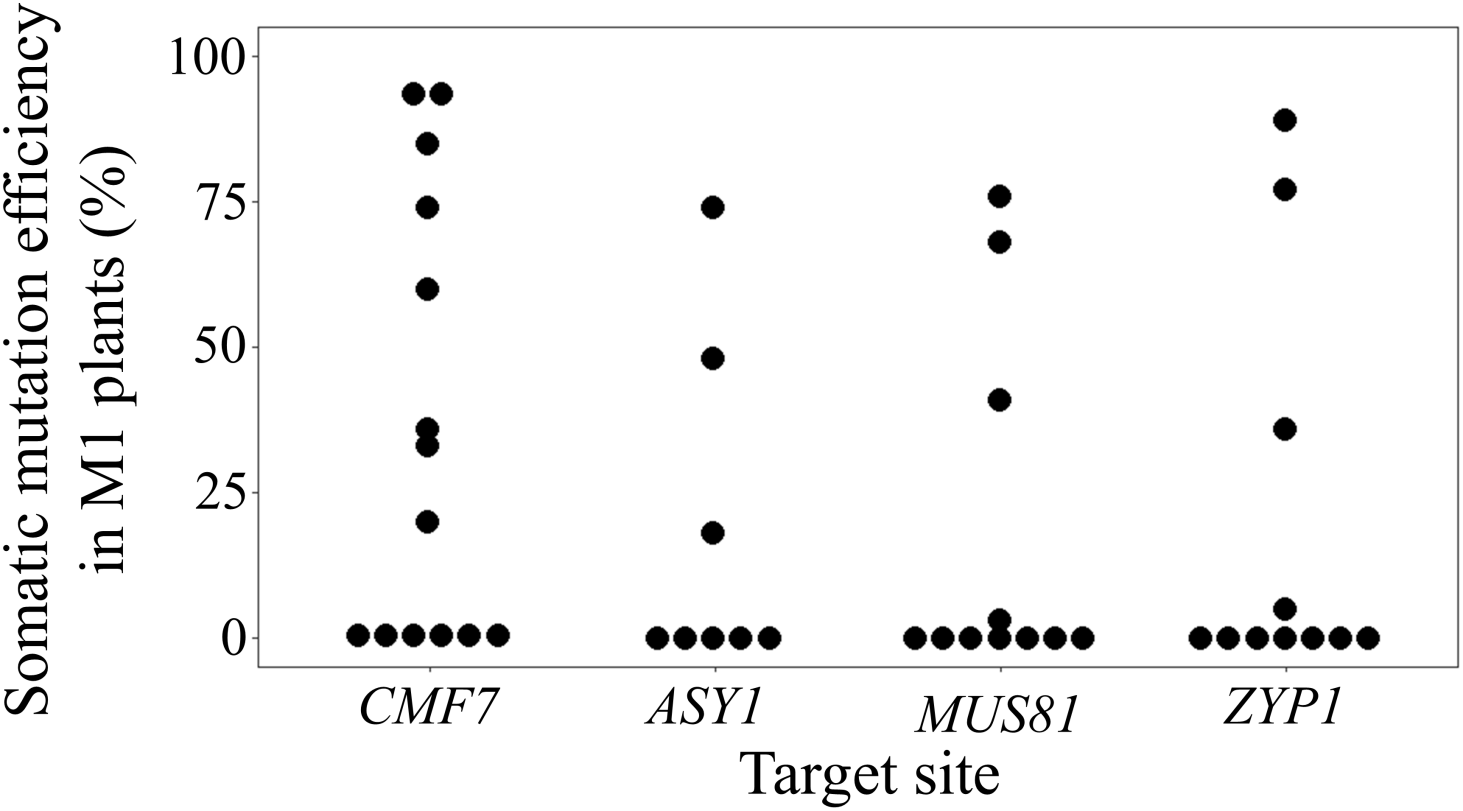
BSMVIGE mediated somatic gene editing in barley. Somatic mutation efficiency at *CMF7*, *ASY1*, *MUS81*, and *ZYP1* target sites 5 wpi in *ZmUBI*::*Cas9* plants inoculated with BSMVIGE-CMF7, -ASY1, -MUS81, and -ZYP1 virus saps, respectively.

To investigate the feasibility of BSMV-mediated genome editing in barley, initially, barley cv. Golden Promise plants that constitutively express *Cas9* driven by the *ZmUBI1* (*maize ubiquitin 1*) promotor were isolated. For a proof-of-principle of BSMVIGE in barley, a Cas9 target motif within the *ALBOSTRIANS* gene, *CMF7*, that had been successfully used for targeted gene editing, was selected (Li et al., 2019). As a negative control, a target motif specific to the *AvGFP* gene (Wang et al., 2017) that is absent in WT barley was chosen. For both target sequences, cognate gRNA 5’-ends were inserted into the BSMV-γ-sg vector resulting in BSMV-γ-sgCMF7 or BSMV-γ-sgGFP. The BSMV infection was performed according to (Yuan et al., 2011). For virus sap production, i.e. BSMVIGE-CMF7 or -GFP sap, *N. benthamiana* was infected with *Agrobacterium* harboring BSMV-α, BSMV-β, and BSMV-γ-sgCMF7 or BSMV-γ-sgGFP, respectively (**Figure 1B**). Then, virus sap was used for rub-infection of fully-emerged second and third leaves of 3-4 weeks old *ZmUBI*::*Cas9* barley plants (**Figures 1C, D**). Assuming that systemic movement of the virus may lead to diverse Cas9-induced mutations in different plant tissues, potential presence of BSMV-mediated editing at the *CMF7* target site was checked across multiple leaf tissues and emerging awns of different tillers in BSMVIGE-CMF7 or -GFP inoculated *ZmUBI*::*Cas9* transgenic barley plants. To do so, pooled tissue samples, collected at 5 weeks post-infection (wpi) from individual plants, were used for Sanger read analysis using the ICE synthego tool to estimate the editing efficiency/types (**Figures 1E, F**) (Conant et al., 2022). Somatic editing was detected at the CMF7 target site in 8 out of 14 *ZmUBI*::*Cas9* plants inoculated with BSMVIGE-CMF7. Mutation efficiencies of up to 94% in an individual plant and a mean mutation efficiency of 35% across all infected plants in two independent experiments were found (**Figure 2**, **Supplementary Table 3**). No editing in the target region was found in any *ZmUBI*::*Cas9* barley plant infected with BSMVIGE-GFP, confirming the specificity of BSMVIGE-induced mutations for the *CMF7* target motif (**Supplementary Table 3**). Given successful somatic editing in 8 out of 14 plants, we asked whether the presence of viral RNA in a given plant positively correlates with somatic editing or whether in turn absence of somatic editing in some plants was due to the absence of the virus. Note, while in wheat or other barley cultivars (Holzberg et al., 2002; Hu et al., 2019) infected with BSMV typical BSMV symptoms including yellow stripes/mosaic leaves are found, neither in BSMV- nor in rub-inoculated (no virus) *ZmUBI*::*Cas9* plants any obvious phenotypic differences compared to uninoculated *ZmUBI*::*Cas9* plants were found. Hence, absence of detectable BSMV symptoms in our materials inhibits visual identification of infected versus non-infected plants. Accordingly, seven randomly *ZmUBI*::*Cas9* plants infected with BSMVIGE-CMF7 with mutation efficiencies ranging from zero to 94% were selected and analyzed for the presence of BSMV RNA 5 wpi. Except for a single plant that showed no signs of editing, BSMV was found in all further analyzed plants which include five plants with editing and one plant without editing. Hence, viral presence is required for editing at the target site but it does not assure *in planta* editing at detectable levels across various parts of a plant 5 wpi.

### 3.2 Heritable editing at the *CMF7* target site

**Figure 3:**
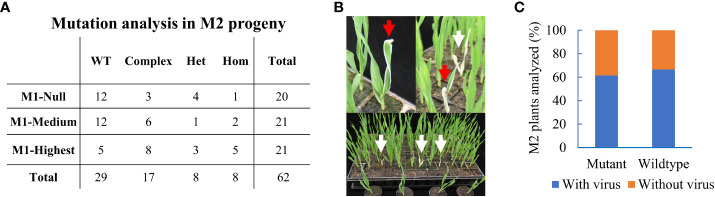
Heritable editing of the *ALBOSTRIANS* locus in barley. **(A)** M2 offspring plants from three selected BSMVIGE-CMF7 M1 plants (M1-Null, M1-Medium, and M1-Highest with somatic mutation efficiencies of 0%, 33%, and 94%, respectively): no mutation at the target locus (wild-type, WT), two or more mutations (complex), one mutant allele (heterozygous, Het) or similar mutation at both alleles (homozygous, Hom); **(B)** M2 progeny showing albino (white arrows) and variegated (red arrows) phenotypes; **(C)** M2 progeny analyzed for mutation at the *CMF7* target site and presence of BSMV. Frequency of mutant or wild-type with presence/absence of BSMV RNA.

Next, we asked whether induced somatic edits were transmitted into offspring plants. Three BSMV-positive primary mutant plants (M1) with variable editing efficiencies of 0, 33, and 94% termed M1-Null, M1-Medium, and M1-Highest, respectively, were selected. The *CMF7* target region was sequenced in at least 20 offspring plants from each of the three selected M1 plants. We speculated that somatic mutation efficiency might aid in setting a selection criterion for choosing the appropriate M1 plant to be mined for heritable editing in offspring. Surprisingly, 33 of 62 analyzed M2 plants (~55%), comprising 8 (n=20), 9 (n=21), and 16 (n=21) M2 offspring from M1-Null, M1-Medium, and M1-Highest, had mutations at the target site (**Figure 3A**). Editing in offspring from M1-Null, being virus-positive five wpi without detectable somatic editing, suggested that BSMV-mediated editing might have occurred after tissue sampling e.g. in reproductive tissues such as the germline or the developing embryos. Hence, our Sanger-based analysis restricted to pooled somatic tissues hampered the identification of BSMV-dependent edits in M1-Null that likely occurred later than five wpi. Among 33 M2 *cmf7* mutants, 17, 8, and 8 offspring plants had complex (bi-allelic or chimeric), heterozygous, and homozygous mutations at the target site, respectively (**Figure 3A**, **Supplementary Table 4**). Three M2 homozygous mutants showed a complete albino phenotype, reflecting a complete *CMF7* loss-of-function phenotype (defective chloroplast development), while 5 M2 homozygous or heterozygous mutants showed a variegated phenotype likely based on residual/hypomorphic CMF7 activity (**Figure 3B**) (Li et al., 2019). Together, BSMVIGE induces somatic mutations at the *CMF7* locus in BSMV-infected *ZmUBI*::*Cas9* barley plants, which can be transmitted to offspring plants.

### 3.3 Virus-free offspring with heritable edits in M2 and M3 generations

BSMV transmission *via* grains depends on the BSMV strain and/or on the host genotype (Carroll, 1972). The generative transmission rate of the BSMVIGE-CMF7 virus (viral RNA presence/absence) was examined in randomly selected M2 offspring plants screened for heritable editing at the *CMF7* target site. Among 38 analyzed M2 plants, 14 were virus-free, comprising 10 mutant and 4 WT plants for the *CMF7* target site, and 24 plants were virus-positive, comprising 16 mutant and 8 WT plants for the *CMF7* target site (**Figure 3C**, **Supplementary Table 5**). Hence, BSMVIGE-CMF7 can be generatively transmitted at a high rate in cv. Golden Promise, while virus-free mutant offspring plants can be recovered.

To determine whether the mutations found in *cmf7* M2 plants will segregate in the M3 generation, a mutation analysis at the target region in M3 progeny from a virus-free M2 mutant plant being heterozygous for an ‘A’ insertion (*cmf7*) was performed. Among nine analyzed M3 offspring, three WT, one homozygous, and five heterozygous plants for *cmf7* were found, suggesting the segregation of the *cmf7* allele in the M3 generation (**Supplementary Table 6**). In addition, whether virus-free M3 *cmf7* mutant progenies can be recovered, for both editing at the *CMF7* target site and BSMV presence/absence in M3 progenies from a virus-positive M2 *cmf7* mutant with multiple editing events (complex) was screened. Four virus-free and four virus-positive M3 offspring plants (n=8) were recovered. Among all these progeny plants, diverse editing events including complex and homozygous mutations at the *CMF7* target site, which were transmitted from the M2 parent, were detected (**Supplementary Table 6**). Thus, regardless of virus transmission, BSMVIGE-induced *cmf7* mutations are inherited into M3 progenies from M2 plants.

### 3.4 BSMVIGE induces somatic editing in putative meiotic genes in barley

To explore the reliability of BSMVIGE in barley, three putative meiosis-related genes that encode for the meiotic chromosome axis-associated protein ASY1 (Armstrong et al., 2002), a dHJ resolvase involved in the class II CO pathway, MUS81 (Hartung et al., 2006), and the transverse filament protein of the SC, ZYP1 (Barakate et al., 2014), were additionally addressed. Cas9 target motifs residing within exon 4 of *ASY1*, exon 1 of *MUS81*, or exon 2 of *ZYP1* were selected and cognate gRNA 5’-ends cloned into BSMV-γ-sg to generate BSMV-γ-sgASY1, BSMV-γ-sgMUS81, and BSMV-γ-sgZYP1 (**Figure 2**, **Supplementary Table 1**). *ZmUBI*::*Cas9* barley plants were infected using BSMVIGE-ASY1, BSMVIGE-MUS81, and BSMVIGE-ZYP1 virus saps. At 5 wpi, 3 out of 8 plants showed mutations at the *ASY1* target site with a single plant showing a maximum mutation efficiency of 74%. In the case of BSMVIGE-MUS81, 3 out of 11 showed editing at the *MUS81* target site while in the case of BSMVIGE-ZYP1, 4 out of 11 plants showed editing at the *ZYP1* target site. The highest mutation efficiency observed in an individual plant at the target site was 76% and 89% for *MUS81* and *ZYP1* target sites, respectively. The mean mutation efficiencies at *ASY1*, *MUS81*, and *ZYP1* target sites were 18%, 17%, and 19%, respectively. In summary, successful somatic editing at three additional target loci in barley suggests BSMV-VIGE as a rapid and reliable genome editing tool in barley.

## 4 Discussion

The use of RNA viruses to deliver gRNAs into plants expressing *Cas9* for genome editing eliminates the need for further stable genetic transformation. Hence, it represents a rather rapid and easy-to-adopt plant genome editing approach, particularly in cereal crops. Therefore, we adapted a robust BSMVIGE workflow in barley that includes Golden Gate-based cloning of a single gRNA into a generic BSMVIGE vector followed by conventional tobacco leaf sap rub-inoculation of *ZmUBI*::*Cas9* barley plants. To estimate somatic editing frequencies throughout infected plants, a rapid Sanger sequencing-based method is used. Accordingly, among a limited number of primary mutant plants, individuals are chosen for heritable editing screening in their offspring. By recreating the *ALBOSTRIANS* barley mutant, initially isolated through X-ray mutagenesis (Hagemann and Scholz, 1962) and *CMF7* being recently identified as the causative gene in barley (Li et al., 2019), we show that virus-mediated heritable targeted gene editing based on BSMVIGE is possible in barley.

Somatic editing at the *CMF7* target site was found 5 wpi in first-generation BSMVIGE-CMF7 virus-infected plants with an average frequency of 57% (n=14; mean mutation efficiency of 35%, ranging from zero to 94%). To possibly define somatic editing frequency as a criterion to choose plants for heritable editing screening in their offspring, M1 plants being positive for BSMV infection with no, intermediate (33%), and highest (94%) mutation efficiencies were selected to screen for heritable editing at the target site in their M2 progeny. Unexpectedly, not only heritable editing in the case of M1-Medium (43% frequency) and M1-Highest (76% frequency) offspring but also in the case of M1-Null (40% frequency) offspring was found. This suggests two non-exclusive possibilities: editing found in M1-Null offspring occurred only after 5 wpi analysis, e.g. in reproductive tissues, embryos or offspring plants, and/or the Sanger-based method used was not sensitive enough to detect the presence of low-frequency mutations. In any case, the highest heritable editing frequency with diverse editing events was found in the M1-Highest offspring. Whether a similar situation is found at other target sites, is unclear. However, M1 plants with rather high mutation efficiencies, represent obvious choices to screen for desired mutants in their offspring.

Notably, BSMV transmission into the next generation was found in barley, similar to wheat (Li et al., 2021), but at a higher frequency. However, virus-free plants with inherited mutations were obtained in both M2 and M3 generations and thus desired materials can be rapidly isolated by screening both for mutations at the target site(s) and viral presence/absence. In addition to our initial target site at the *CMF7* gene, induction of somatic editing was also achieved at three putative meiosis-related genes, *ASY1*, *MUS81*, and *ZYP1*, with frequencies of 38%, 27%, and 36% (n=11), respectively, suggesting broad applicability of BSMVIGE for targeted genome engineering in barley.

The fusion of tRNAs or mobile RNA elements such as flowering locus T (FT) to gRNAs delivered by TRV can increase the heritable editing frequency in *A. thaliana* (Nagalakshmi et al., 2022) and *N. benthamiana* (Ellison et al., 2020). In wheat, both increased and decreased heritability of induced mutations upon fusion of RNA elements to gRNAs in BSMV were reported (Li et al., 2021; Chen et al., 2022a; Wang et al., 2022). These contradictory findings might be attributed to the different lengths and types of fusion RNAs as well as the different wheat genotypes employed. Whether in barley cv. Golden Promise the addition of tRNA or mobile RNA sequences to gRNAs improves BSMV-mediated (heritable) editing at target sites, should be addressed in future studies.

Multiple gRNAs addressing different targets can be expressed in a single virus such as TRV (Ellison et al., 2020) or *Potato virus* X (Uranga et al., 2021). Considering the limited cargo capacity of BSMV (Bruun-Rasmussen et al., 2007), stacking up gRNA arrays seems unfeasible. However, successful multiplexed gene editing in wheat using a mixed *Agrobacterium* pool strategy based on BSMVIGE (Li et al., 2021; Chen et al., 2022a; Wang et al., 2022), paves the way to test in the future, whether a similar strategy could also be adopted for barley. Furthermore, in existing mutant plants isolated either by conventional transformation strategies or by BSMVIGE being virus-free, likely BSMVIGE could be harnessed to edit independent genes of interest.

The current BSMVIGE approach is restricted to available barley genotypes that stably express *Cas9*, which is cv. Golden Promise in the present investigation. However, in addition to the possibility of generating further barley cultivars stably expressing *Cas9*, the current *Cas9* transgene may be introgressed into other cultivars of interest (Chen et al., 2022a; Wang et al., 2022). Further, as demonstrated for wheat (Budhagatapalli et al., 2020), the development of *Cas9* expressing haploid inducer barley lines infected with BSMV carrying gRNA(s) of interest could be utilized to induce mutations in the genome of the desired genotypes. Recently, a modified four-component BSMV system with increased cargo capacity (Cheuk and Houde, 2018) enabled transient somatic editing in cotton using split SpCas9 (Chen et al., 2022b). Whether reliable and frequent heritable editing based on a similar strategy can be achieved, needs to be addressed. Given the broad host range of BSMV and the application of BSMVIGE in barley (this study) or wheat (Li et al., 2021; Chen et al., 2022a; Wang et al., 2022), likely BSMVIGE can be adopted for other crops such as oat (Cho et al., 1999; Zhang et al., 1999) or rye (Popelka and Altpeter, 2003), where genetic transformation procedures are available.

## Data availability statement

The original contributions presented in the study are included in the article/**Supplementary Material**. Further inquiries can be directed to the corresponding author.

## Author contributions

SHe acquired funding and designed this study. ST-N-A supported by FH, JL, RD and SD conducted most of the experiments and analyzed the data. SHi, GH, and JK generated barley *Cas9*-expressing lines. ST-N-A and SHe drafted the manuscript. All authors contributed to the article and approved the submitted version.

## Supplementary material

Supplementary Figure 1 Sanger chromatogram analysis using ICE synthego tool (Conant et al., 2022) reveals somatic editing at target sites. Sanger traces at target sites within *CMF7* **(A)**, *ASY1* **(B)**, *MUS81* **(C)** and *ZYP1* **(D)** of selected BSMV-inoculated (top panel) compared with BSMV-uninoculated (bottom panel) M1 plants showing high, intermediate or no somatic editing 5 wpi analysed by ICE synthego tool. Details on plant materials indicated are given in **Supplementary Table 3**. In each case, the 20 bp Cas9 target motif is underlined in bold lines, while PAM is highlighted using dotted lines.
