# Supplementary figures and images for: Barley stripe mosaic virus-mediated somatic and heritable gene editing in barley (Hordeum vulgare L.)
Source: Front Plant Sci. 2023 Jun 19;14:1201446. doi: 10.3389/fpls.2023.1201446 (PMC10315673; doi:10.3389/fpls.2023.1201446)

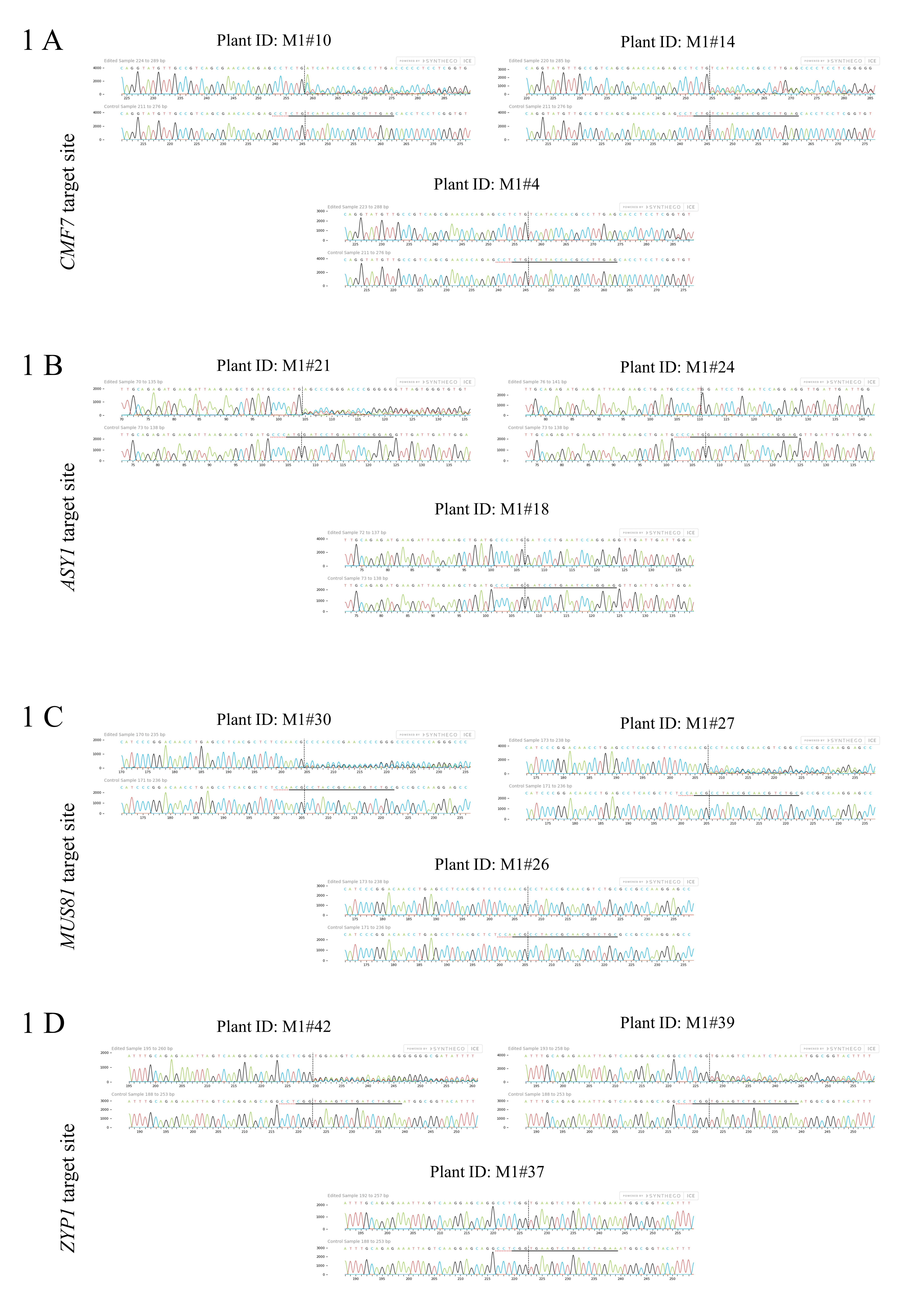

Supplement: Supplementary Figure 1 — Sanger chromatogram analysis using ICE synthego tool (Conant et al., 2022) reveals somatic editing at target sites. Sanger traces at target sites within CMF7 (A), ASY1 (B), MUS81 (C) and ZYP1 (D) of selected BSMV-inoculated (top panel) compared with BSMV-uninoculated (bottom panel) M1 plants showing high, intermediate or no somatic editing 5 wpi analysed by ICE synthego tool. Details on plant materials indicated are given in Supplementary Table 3 . In each case, the 20 bp Cas9 target motif is underlined in bold lines, while PAM is highlighted using dotted lines. [file Image_1.tif]
